# Frequently arising ESX-1-associated phase variants influence *Mycobacterium tuberculosis* fitness in the presence of host and antibiotic pressures

**DOI:** 10.1128/mbio.03762-24

**Published:** 2025-01-28

**Authors:** Michael J. Luna, Peter O. Oluoch, Jiazheng Miao, Peter Culviner, Kadamba Papavinasasundaram, Eleni Jaecklein, Scarlet S. Shell, Thomas R. Ioerger, Sarah M. Fortune, Maha R. Farhat, Christopher M. Sassetti

**Affiliations:** 1 Department of Microbiology, UMass Chan Medical School 200789 , Worcester, Massachusetts, USA; 2 Department of Biomedical Informatics, Harvard Medical School 12262 , Boston, Massachusetts, USA; 3 Department of Immunology and Infectious Diseases, Harvard TH Chan School of Public Health 1811 , Boston, Massachusetts, USA; 4 Department of Biology and Biotechnology, Worcester Polytechnic Institute 1857 , Worcester, Massachusetts, USA; 5 Department of Computer Science and Engineering, Texas A&M University 8718 , College Station, Texas, USA; 6 Pulmonary and Critical Care Medicine, Massachusetts General Hospital 14736 , Boston, Massachusetts, USA; Washington University in St. Louis School of Medicine, St. Louis, Missouri, USA

**Keywords:** tuberculosis, bacterial genetics, phase variation

## Abstract

*Mycobacterium tuberculosis* (Mtb) exhibits an impressive ability to adapt to rapidly changing environments, despite its genome’s apparent stability. Recently, phase variation through indel formation in homopolymeric tracts (HT) has emerged as a potentially important mechanism promoting adaptation in Mtb. This study examines the impact of common phase variants associated with the ESX-1 type VII secretion system, focusing on a highly variable HT upstream of the ESX-1 regulatory factor, *espR*. By engineering this frequently observed indel into an isogenic background, we demonstrate that a single nucleotide insertion in the *espR* 5′UTR causes post-transcriptional upregulation of EspR protein abundance and corresponding alterations in the EspR regulon. Consequently, this mutation increases the expression of ESX-1 components in the *espACD* operon and enhances ESX-1 substrate secretion. We find that this indel specifically increases isoniazid resistance without impacting the effectiveness of other drugs tested. Furthermore, we show that two distinct observed HT indels that regulate either *espR* translation or *espACD* transcription increase bacterial fitness in a mouse infection model. The presence of multiple ESX-1-associated HTs provides a mechanism to combinatorially tune protein secretion, drug sensitivity, and host-pathogen interactions. More broadly, these findings support emerging data that Mtb utilizes HT-mediated phase variation to direct genetic variation to certain sites across the genome in order to adapt to changing pressures.

**IMPORTANCE:**

*Mycobacterium tuberculosis* (Mtb) is responsible for more deaths worldwide than any other single infectious agent. Understanding how this pathogen adapts to the varied environmental pressures imposed by host immunity and antibiotics has important implications for the design of more effective therapies. In this work, we show that the genome of Mtb contains multiple contingency loci that control the activity of the ESX-1 secretion system, which is critical for interactions with the host. These loci consist of homopolymeric DNA tracts in gene regulatory regions that are subject to high-frequency reversible variation and act to tune the activity of ESX-1. We find that variation at these sites increases the fitness of Mtb in the presence of antibiotic and/or during infection. These findings indicate that Mtb has the ability to diversify its genome in specific sites to create subpopulations of cells that are preadapted to new conditions.

## INTRODUCTION

Microorganisms have adopted a wide variety of mechanisms for generating genetic variation, ranging from the use of error-prone polymerases to a myriad of means of horizontal gene transfer (1–4). For the majority of prokaryotes, horizontal gene transfer is a significant driver of genetic diversity, and this sharing of genetic material is perhaps the most crucial mechanism for adapting to changing environmental pressures (5–7). *Mycobacterium tuberculosis* (Mtb) stands out among bacterial pathogens in that this species does not acquire new genetic material by way of horizontal gene transfer (HGT) (8, 9). Despite the resulting stability of its genome, Mtb displays a tremendous amount of phenotypic diversity, having adapted to various host populations, epidemiological contexts, and drug pressures (10, 11). Although there is evidence of HGT in the evolutionary history of Mtb (12) and recent studies indicate that this species has retained some HGT capability (13), Mtb likely evolved by clonal expansion and the diversification of its genome occurs primarily via *de novo* mutation (14). Although these rare and random mutations can alter antibiotic susceptibility, transmission rates, and disease severity (15–17), this relatively slow evolutionary process is likely insufficient to allow adaption to rapidly changing environments (15, 16, 18).

Another genetic mechanism adopted by some bacteria to ensure rapid adaptation to environmental shifts is the deployment of contingency loci that target high-frequency genetic variation to specific sites in the genome. Although the mutagenic mechanisms vary, homopolymeric tracts (HT) and other simple sequence repeats are common sites for this process (19). These repetitive regions exhibit relatively high rates of insertions and deletions (indels) relative to the rest of the genome due to slipped strand mispairing during replication (20, 21). If these repeats are found within the coding body of a gene, indels may alter or inactivate the encoded protein, whereas indels in regulatory regions can modulate expression levels (20–23). This process can create substantial subpopulations of cells that are pre-adapted to frequently encountered stresses. Importantly, the high mutation rate also facilitates reversion of indels at these loci, restoring the original phenotype if it once again becomes advantageous (22, 24). This process of targeted and reversible high-frequency mutation has been coined phase variation (20, 21, 24). Pathogenic bacteria make extensive use of phase variation to avoid immune recognition, both by toggling the expression of single antigens (23) or by combinatorially layering repetitive regions within multiple genes dedicated to the synthesis and modification of surface polysaccharides (25). This strategy also appears to play a major role in the adaptation to antibiotic pressure (26).

Multiple recent reports indicate that Mtb takes advantage of homopolymer-mediated phase variation to adapt to stress (27, 28). The first example of this process to be described in Mtb occurs at the *glpK* locus, which encodes the glycerol-3 kinase necessary for glycerol assimilation. This gene contains a seven base pair poly-G tract, a structure that is highly mutagenic in mycobacteria (29, 30). This homopolymer is a common site of gene-inactivating, frameshifting indels in clinical isolates. *glpK* phase variants are strongly associated with drug resistance in natural isolates, and the loss of functional GlpK decreases sensitivity to multiple drugs, both *in vitro* and in mouse models of therapy (27, 28). In contrast, an intact *glpK* gene has been shown to increase the fitness of the bacterium within certain host environments (31, 32). Thus, the Mtb *glpK* locus fulfills the canonical role of a phase variable gene, as both the wild-type and mutant alleles are required for optimal fitness under different conditions, and the bacterial population can relatively rapidly switch between these phenotypic states via frequent homopolymer-associated indels.

In addition to *glpK*, phylogenomic analyses of a large collection of clinical isolates identified indels in 44 homopolymeric tracts that occurred at a high frequency, suggesting that they too are under positive selection (33). Strikingly, a number of these variable homopolymeric tracts were found to be associated with the ESX-1 type VII secretion system, a major virulence determinant in Mtb that plays a complex role during infection (34–38). This system not only promotes the fitness of the bacterium by supporting intracellular growth and promoting cell wall integrity but is also responsible for the secretion of immunodominant antigens that could lead to immune restriction of bacterial growth (36, 39, 40). These potentially antagonistic functions suggest a possible benefit of tuning ESX-1 function across different host environments. Components of the ESX-1 system are encoded in two distinct loci. The majority of the structural components are encoded at the RD1 locus, the deletion of which is largely responsible for attenuation of the live BCG vaccine (36, 37). In addition to this locus, the distally encoded *espACD* operon encodes components necessary for the secretion of protein substrates, among which is the ESAT-6/CFP-10 heterodimer (40, 41). This operon is regulated by multiple inputs (42–44), including the nucleoid-associated protein EspR, whose regulon includes a number of other targets, most notably among lipid metabolic genes (45, 46). We previously described clinically prevalent single-nucleotide indels in three different HTs that are in or near genes related to ESX-1. One polyA HT is near the promoter of *espACD* at a site predicted to interact with the C-terminal domain of RNA polymerase, and the prevalent variant removes a single base pair from this HT and reduces the transcription of the operon (33). A second HT indel causes frameshift mutations in the gene encoding the chaperone EspK and is predicted to inactivate the gene. A third variable HT is upstream of *espR*.

In this work, we characterize the consequences of the common single base pair insertion in the HT upstream of *espR*. We demonstrate that this indel frequently arises across diverse lineages of Mtb and found it to impact EspR translation by modifying its 5′UTR structure. This mutation increases expression of *espACD* and enhances ESX-1 secretory activity. We then demonstrate that HT indels upstream of *espACD* and *espR* increase fitness in the presence of isoniazid and/or during infection of mouse lungs, suggesting that the combinatorial effects of multiple phase variable sites could mediate adaptation to distinct selective pressures.

## RESULTS

### Homopolymer indels upstream of *espR* arise frequently in Mtb clinical isolates

**Fig 1:**
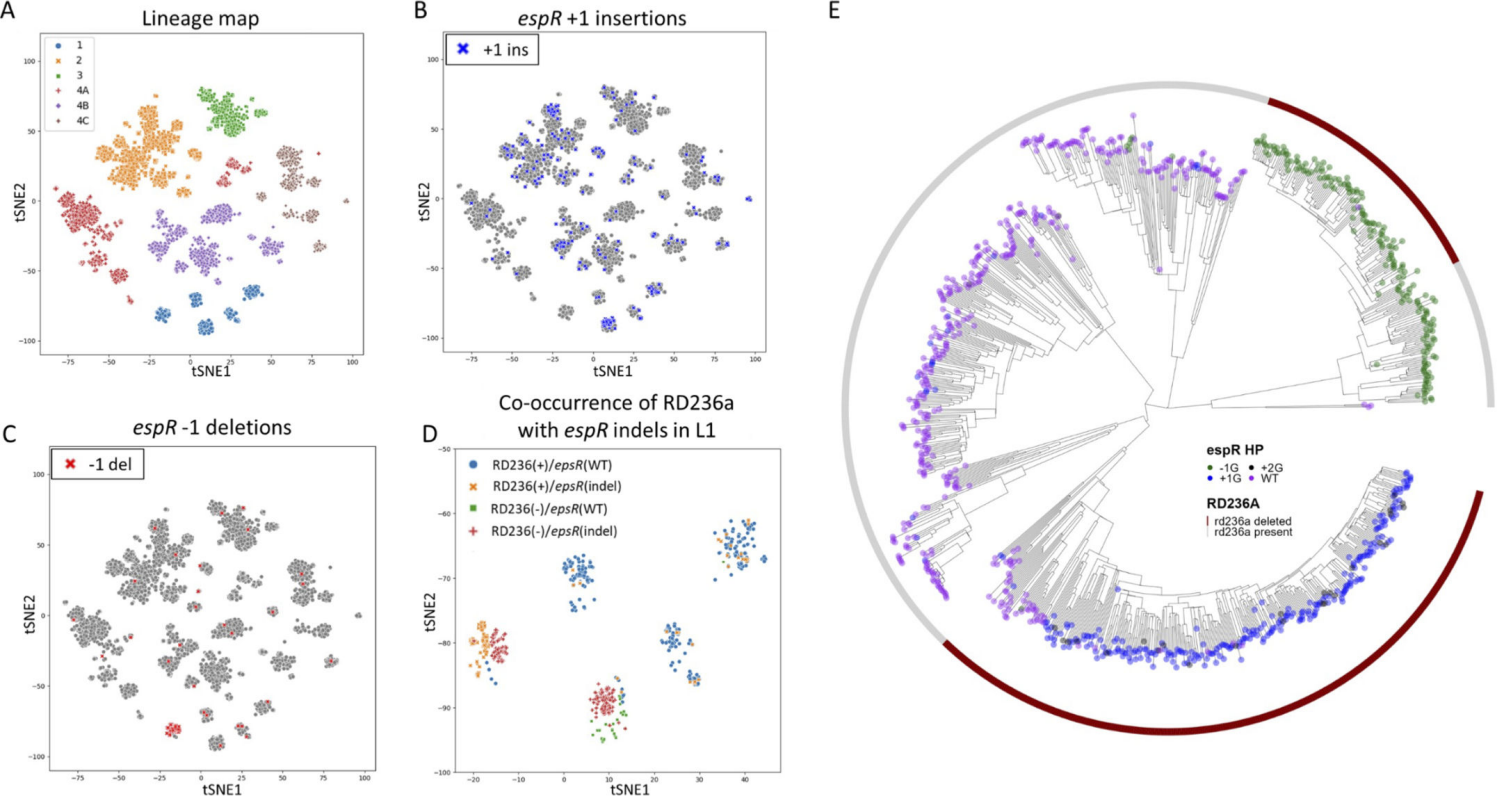
*espR* homopolymer indels frequently occur across diverse clinical isolates. (**A–D**) tSNE plots generated from 26,903 clinical Mtb isolates. (**A**) All isolates colored according to lineage. L1 in blue, L2 in orange, L3 in green, L4A in red, L4B in purple, and L4C in brown. (B and C) All isolates colored by *espR* homopolymer genotype with (B) homopolymer +1 insertions marked in blue, or (C) homopolymer −1 deletions marked in red, (D) L1 isolates colored by both RD236a genotype and *espR* homopolymer genotype. Isolates marked blue have an intact RD236a locus and are WT at the *espR* homopolymer locus. Isolates marked in orange have an intact RD236a locus with an indel at the *espR* homopolymer locus. Isolates marked in green have a deleted RD236a locus, with a WT *espR* locus. Isolates marked in red have an RD236a deletion and an *espR* homopolymer indel. (E) A core SNP-based phylogenetic tree of lineage 1 strains (933 publicly available L1 strains predominantly from Vietnam and India). Outer ring represents whether RD236a is present (gray) or deleted (red). Tree tips are colored according to genotype at the *espR* homopolymer locus. Tree is rooted using *Mycobacterium canettii* as outgroup.

We previously noted frequently occurring indels within a homopolymeric tract 144 bp upstream of *espR* in a relatively large (265 bp) intergenic region (33). To visualize how these mutations were phylogenetically distributed, we leveraged a set of 26,903 clinical Mtb isolates including four major Mtb lineages (Fig. 1A). Across all lineages investigated, both insertions (Fig. 1B) and deletions (Fig. 1C) within this homopolymer frequently arose independently, suggesting that they may be positively selected. Although these indels were well distributed across the most lineages, clustering of the mutations was observed among a subset of lineage 1 (L1) isolates. Upon closer examination of these L1 strains, we observed an association between *espR* homopolymer indels and a large L1-restricted deletion upstream of the *espACD* operon known as RD236a (Fig. 1D) (47). To better visualize the population structure of these L1 strains, we built a phylogenetic tree from a subset of L1 strains and observed an association between *espR* homopolymer indels and RD236a in two subclades (Fig. 1E). One subclade contained an ancestral RD236a event and +1 *espR* indels emerged later in this background to become the predominant allele. The other subclade contained an ancestral −1 *espR* indel, followed by an RD236a deletion. The remaining L1 subclades resembled other lineages where the reference 7G *espR* allele predominates. Further variants and reversions emerged throughout the tree. As RD236a removes multiple EspR binding sites upstream of *espA* (48), the apparent epigenetic relationship between this deletion and *espR* HT indels (39) suggested that the *espR* mutations may alter the expression of *espACD*.

### A common single base pair HT expansion upstream of *espR* causes transcriptional changes in the *espR* regulon

**Fig 2:**
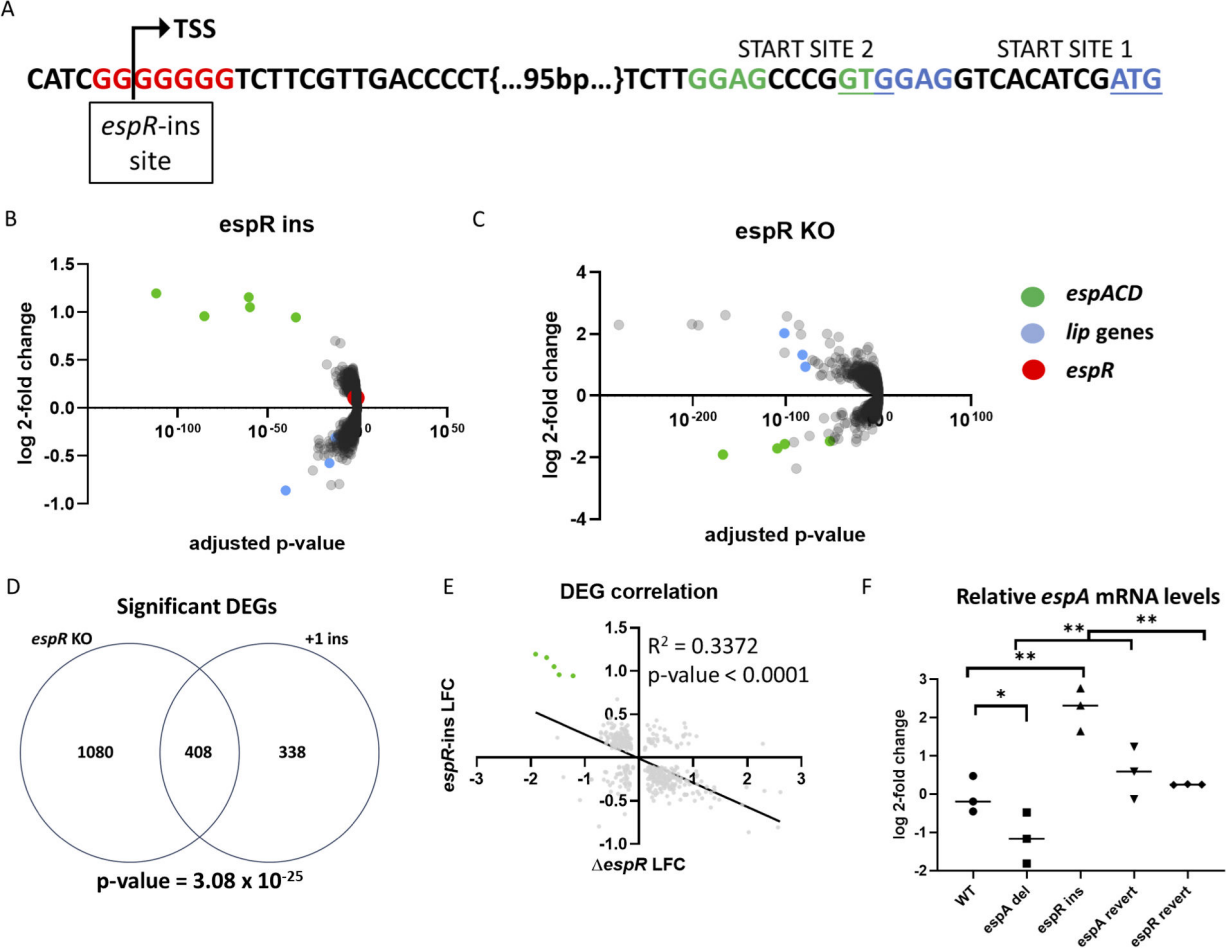
*espR* upstream insertion results in upregulation of EspR regulon. (A) Schematic showing the variable 7G homopolymer upstream of *espR*. The homopolymer begins 144 bp upstream of the *espR* translational start site. The predicted transcriptional start site rests within the homopolymer, with the wild-type allele predicted to include a 5G stretch at the 5′ end of the UTR. The canonical translational start site and accompanying ribosomal binding site are labeled as START SITE 1, with a second putative start codon and associated ribosomal binding site labeled START SITE 2, with start codons underlined. (**B and C**) Volcano plots showing RNAseq results from (**B**) *espR* ins variant and (**C**) ∆*espR*. Genes found in the *espACD* operon are highlighted in green, select *lip* genes in blue, and *espR* in red. (**D**) Venn diagram showing the overlap of all significantly differentially expressed genes (*P*-adj ≤ 0.05) in both mutants according to DeSeq2, with the significance of the overlap calculated using a hypergeometric test. (**E**) Correlation plot showing all genes significantly dysregulated in both mutants with *espACD* operon highlighted in green. (**F**) qPCR showing changes in relative *espA* expression, normalized to *sigA* levels and compared using one-way ANOVA followed by a Holm-Sidak test. Points indicate individual biological replicates, and the median of these values is shown. *P*-value: *, <0.05; **, <0.01.

The most commonly observed *espR*-adjacent mutation was a single base pair expansion in the HT that begins 144 base pairs upstream of the initiation codon, overlapping the previously determined transcriptional start site (Fig. 2A) (49). We first tested whether this variant had any regulatory consequences by using oligonucleotide-based recombineering to engineer this “*espR*-ins” variant into the laboratory Mtb strain H37Rv (50) and comparing its transcriptional profile with the parental strain. This RNAseq study identified 746 differentially expressed genes (DEGs) between mutant and wild type (*P*-adj ≤ 0.05) (Fig. 2B; Table S1), in line with previous reports that EspR modulates an extensive regulon (45, 51). The five genes with the largest magnitude of change between wild type and mutant were the five members of the *espACD* operon. Each of these genes was similarly induced by approximately twofold in the mutant relative to wild type, as expected from the operonic structure of this locus. Among the most strongly downregulated genes were lipid esterases with known EspR binding sites, such as *lipU*, *lipL*, and *lipF* (45). Interestingly, this mutation produced no significant dysregulation of the *espR* mRNA itself.

To more rigorously associate these transcriptional effects with EspR, we compared the mRNA profile of the *espR*-ins strain to that of a newly constructed *espR* knockout mutant (∆*espR*) (Fig. 2C; Table S2). Comparing the differentially expressed genes between the ∆*espR* and *espR*-ins strains suggested that the natural variant conferred a hypermorphic *espR* phenotype. Many of the same genes were dysregulated in both mutants, but with the directionality reversed. For instance, the *espACD* operon was downregulated nearly fourfold in the knockout compared to the twofold upregulation seen in the *espR*-ins mutant. In total, 1,488 genes were significantly dysregulated in the ∆*espR* strain, and the expression of 408 of these was also altered by the *espR*-ins mutation (Fig. 2D). This overlap represents a statistically significant enrichment (*P* = 3.08 × 10^−25^). Moreover, comparing the expression level of all DEGs in each mutant background revealed a significant negative correlation and highlighted the preferential effect of *espR* mutations on the *espACD* locus (*P* < 0.0001) (Fig. 2E).

To confirm that the transcriptional differences between the wild-type and *espR*-ins strains were caused by the engineered mutation, we again used recombineering to revert this mutation back to the wild-type sequence, creating the *espR*-rev strain. In parallel, we reverted the −1 indel in the *espA* promoter region, that we previously reported to repress *espACD* transcription (33). Using these strains, we confirmed that both the induction of *espA* by the *espR*-ins mutation and the repression of *espA* by the *espA*-del mutation reverted back to wild-type levels by restoring the original sequence at each chromosomal site (Fig. 2F).

### The *espR*-ins variant acts at the post-transcriptional level to increase the secretion of ESX-1 substrates

**Fig 3:**
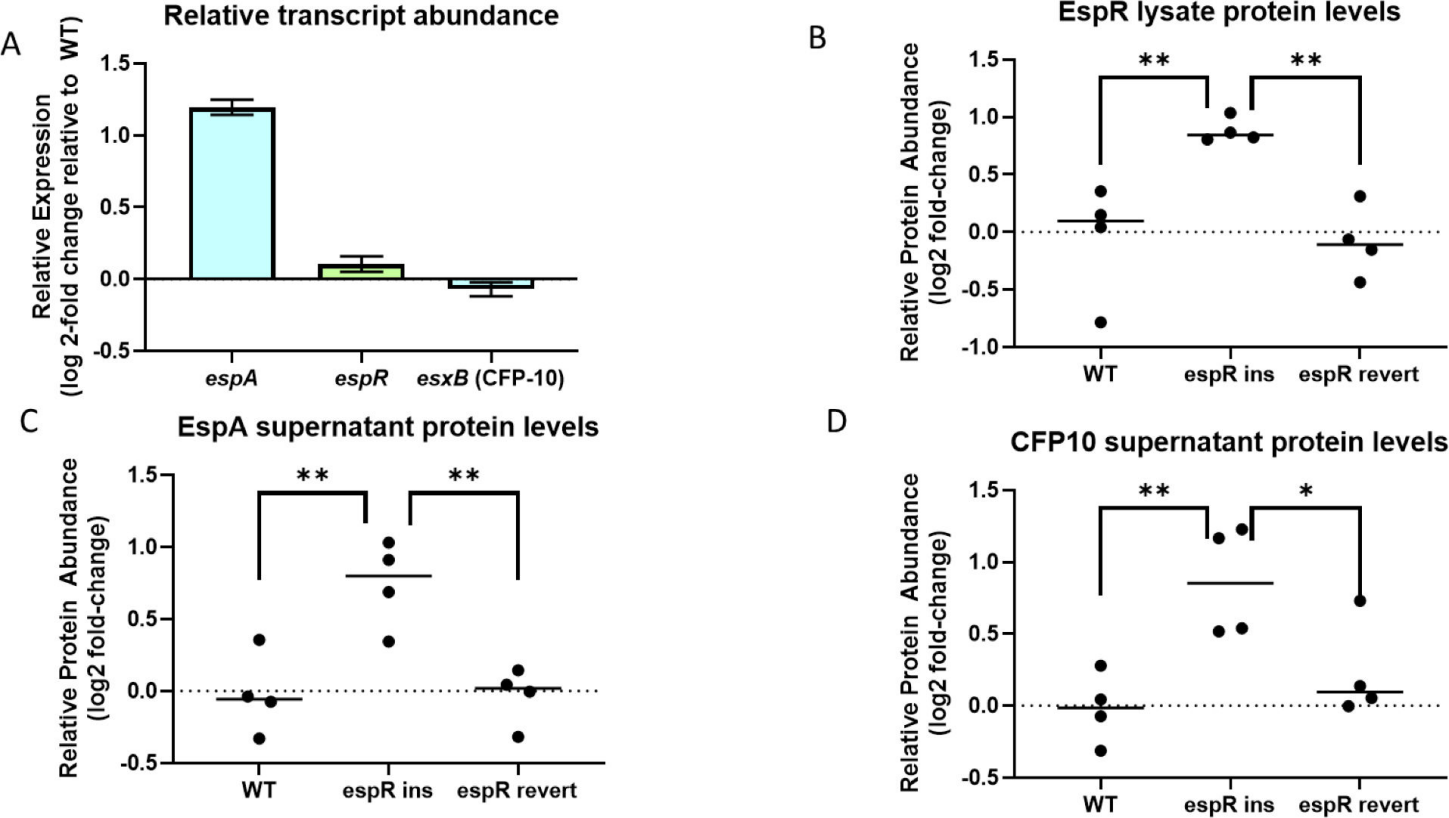
*espR*-ins mutants result in changes at the protein level. (A) mRNA levels observed in RNAseq for *espA*, *espR*, and *esxB* (CFP-10) in the *espR* homopolymer mutant strain. Error bars denote standard error. (**B–D**) Mass spectrometry was performed to evaluate EspR, EspA, and CFP-10 protein levels, respectively. Individual points represent biological replicates, with median value marked as a line. Values were normalized to SigA levels for each sample. Individual points represent four biological replicates with median indicated by line. Samples were compared using ANOVA followed by Dunnett’s multiple comparison test. *P*-value: *, <0.05; **, <0.01.

The *espR*-ins variant is located in the 5′UTR of the gene and alters the expression of downstream *espR* targets without affecting the abundance of *espR* mRNA (Fig. 3A). Based on these data, we hypothesized that this variant has a post-transcriptional effect on EspR protein levels. To test this hypothesis and assess the functional consequences of the variant on ESX-1 activity, we used targeted mass spectrometry to quantify the abundance of relevant proteins in cell lysates or supernatants. Consistent with a post-transcriptional effect on EspR expression, we found that lysates of the *espR*-ins strain contained significantly more EspR protein than the wild type (Fig. 3B). Similarly, EspA levels were increased in the supernatant of the *espR*-ins strain (Fig. 3C), which corresponded to the observed increase in *espACD* mRNA. To more specifically assess ESX-1 function, we quantified the secretion of the CFP-10 protein, an ESX-1 substrate whose mRNA levels are not affected by the *espR*-ins variant. We noted a significant increase in CFP-10 abundance in the supernatants of the *espR*-ins strain (Fig. 3D), suggesting that the increased expression of *espR* and *espACD* resulted in an equivalent increase in protein secretion by ESX-1. The abundances of all three proteins returned to wild-type levels in the *espR*-rev strain, indicating that these differences were caused by the engineered variant.

### The *espR*-ins variant relieves 5′UTR-dependent repression of protein expression

**Fig 4:**
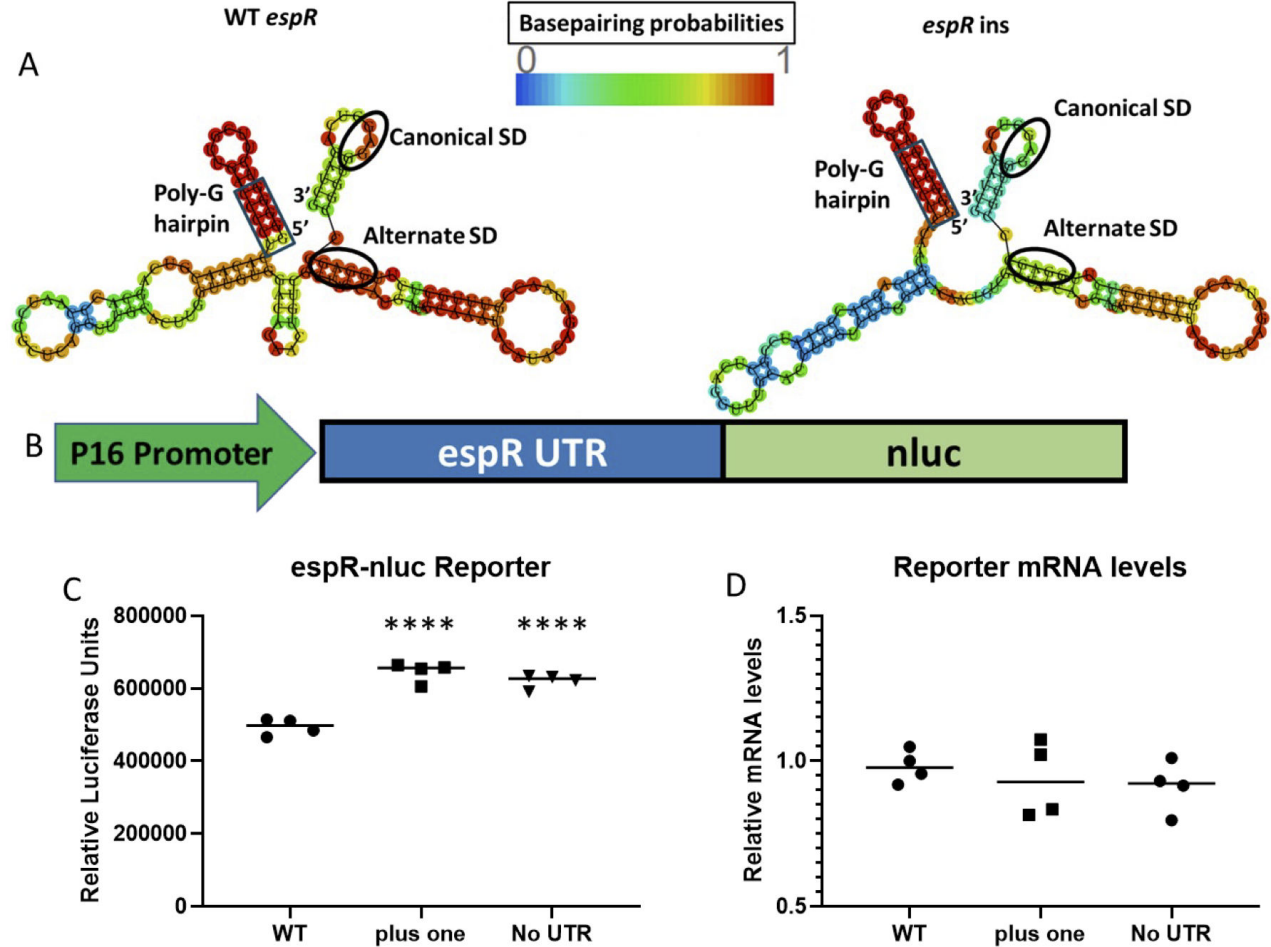
*espR*-ins results in a differentially folded 5′UTR and increased translational efficiency. (A) Folding prediction of WT and +1 *espR* untranslated region with color denoting the probability of base pairing at each base. The poly-G motif is enclosed with a box at the 5′ end of the transcript, and two putative Shine-Dalgarno sequences are circled. (**B**) Schematic of *espR* translational reporter. We fused synthetic promoter p16 to the predicted *espR* 5′UTR from TSS to start codon, driving a nanoluc reporter. (**C**) Luminescence of constructs containing WT, +1, and no UTR were measured and compared by ANOVA with Dunnett’s multiple corrections test (D) mRNA levels of nanoluc were compared by qPCR and normalized to *sigA* as an endogenous control and then compared by ANOVA with Dunnett’s multiple corrections test. Points indicate individual biological replicates, and the median of these values is shown. *P*-value: *, <0.05; **, <0.01; ***, <0.001; ****, <0.0001.

The discrepancy between the effect of the *espR*-ins variant on *espR* mRNA and protein levels suggested that this 5′UTR variant could alter EspR translation. To investigate this, we computationally determined the predicted mRNA structure of the 5′ UTR in both the wild-type and mutant species (Fig. 4A). The *espR*-ins variant increased the length of a G/C hairpin at the 5′ end of the UTR, resulting in reduced base pairing probabilities for each of the other predicted hairpins, including sequences corresponding to the predicted ribosome binding site, as well as a second potential alternative Shine-Dalgarno sequence, located at −13 and −23 from the start codon, respectively (Fig. 2A). This model suggests that the insertion of an extra G in the homopolymer may increase ribosomal binding and facilitate more efficient translation.

To experimentally test this prediction, we constructed a translational reporter by fusing the *espR* 5′UTR, with or without the *espR*-ins mutation, to the nanoluciferase (nLuc) open reading frame (Fig. 4B). To control the rate of transcription, these two constructs were both driven by identical synthetic promoters. After transforming these constructs into Mtb, we quantified nLuc abundance using a luciferase assay. Consistent with our mass spectrometry findings, the *espR*-ins variant exhibited significantly higher levels of luminescence (Fig. 4C). In contrast, we detected no differences in nLuc mRNA levels between the two strains by qPCR (Fig. 4D). A similar increase in luminescence was also observed using a construct lacking an extended 5′UTR, containing only its predicted native RBS. Together, these data suggest a repressive regulatory role for the *espR* 5′UTR and indicate that the homopolymer expansion acts to relieve the negative regulation.

### *espR* overexpression decreases isoniazid efficacy

**Fig 5:**
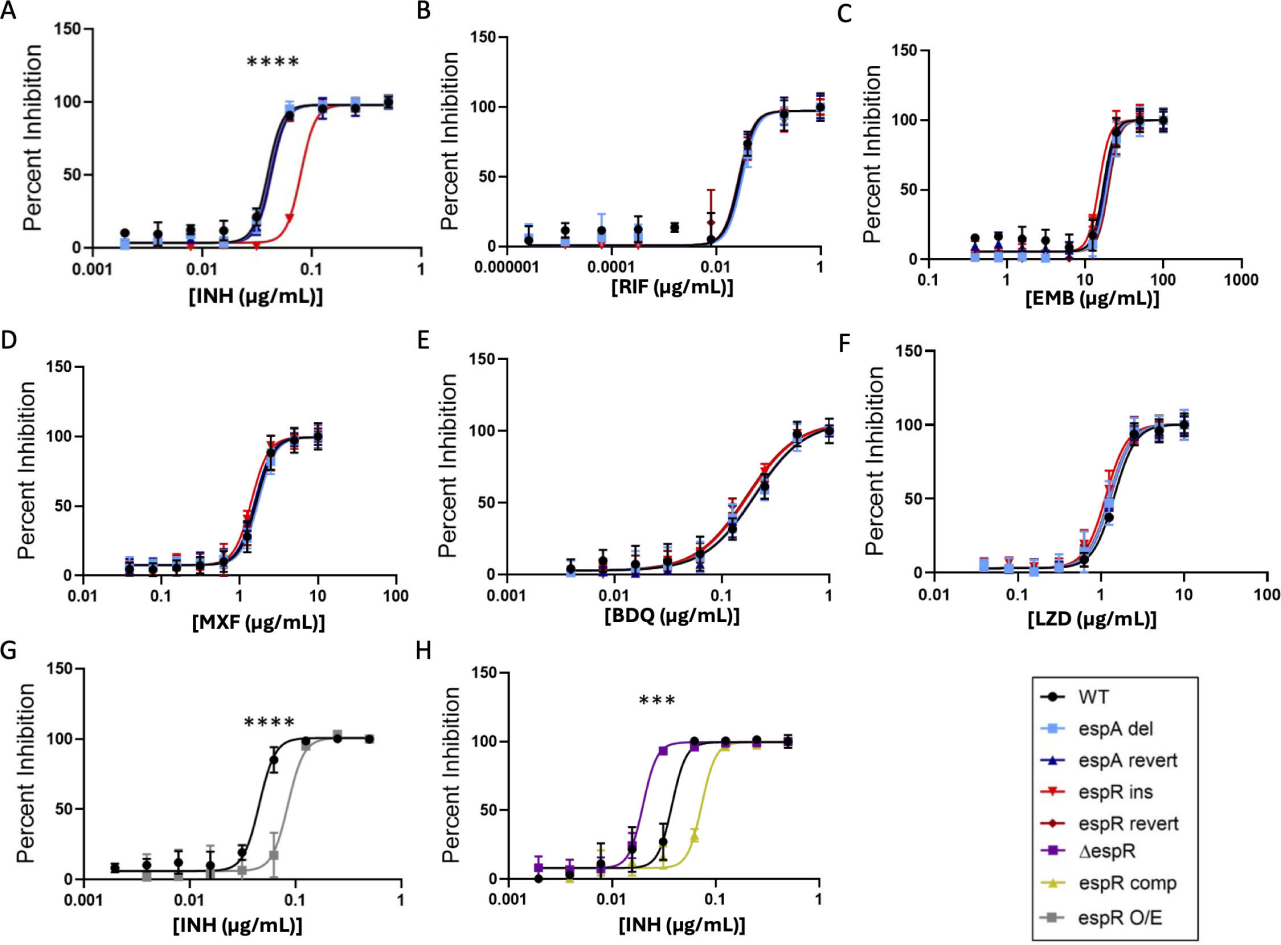
*espR* overexpression leads to an increase in isoniazid MIC. (A–F) Minimum inhibitory concentration curves showing percent inhibition of mutant strains and revertants in the presence of (A) isoniazid, (**B**) rifampicin, (**C**) ethambutol, (**D**) moxifloxacin, (**E**) bedaquiline, or (F) linezolid. Curves were generated using nonlinear regression comparing IC50 values while constraining all other parameters using GraphPad Prism 8. Individual IC50 values were then compared using ANOVA followed by Dunnett’s test when applicable. An additional Bonferroni correction was then performed to account for the number of antibiotics tested. (**G**) MIC curves comparing WT to an *espR* overexpression strain in the presence of isoniazid. After generating curves, IC50 values were compared by *t*-test. (**H**) MIC curves comparing WT, *espR* KO, and complemented *espR* strains. After generating curves from three biological replicates, IC50 values were compared using one-way ANOVA followed by Tukey’s post-test: *P*-value: *, <0.05; **, <0.01; ***, <0.001; ****, <0.0001.

Both *espA*-del and *espR*-ins natural variants appear to be positively selected in clinical isolates (33), but the pressures leading to this selection remained unclear. Therefore, we used both of these mutant strains in parallel to explore potential sources of selection that have led to the frequent emergence of these mutations. Perhaps the strongest pressure that Mtb faces in the modern world is that of antibiotics. As a result, we investigated whether either of these prevalent ESX-associated homopolymer variants resulted in detectable levels of *in vitro* drug resistance. We focused our investigations on six of the most important and commonly administered anti-tubercular drugs (52). Neither of these mutations impacted the minimal inhibitory concentration (MIC) of rifampicin, ethambutol, bedaquline, linezolid, or moxifloxacin. However, the *espR*-ins mutation resulted in a reproducible twofold increase in isoniazid resistance, which was reversed in the *espR*-rev strain (Fig. 5A through F). Consistent with the idea that this mutation acts as a hypermorph, overexpressing an extra copy *espR* phenocopied the *espR*-ins mutation (Fig. 5G), while the ∆*espR* strain displayed an increased isoniazid susceptibility (Fig. 5H).

### ESX-1 associated homopolymeric indels do not detectably alter macrophage cytokine profiles or cell death

**Fig 6:**
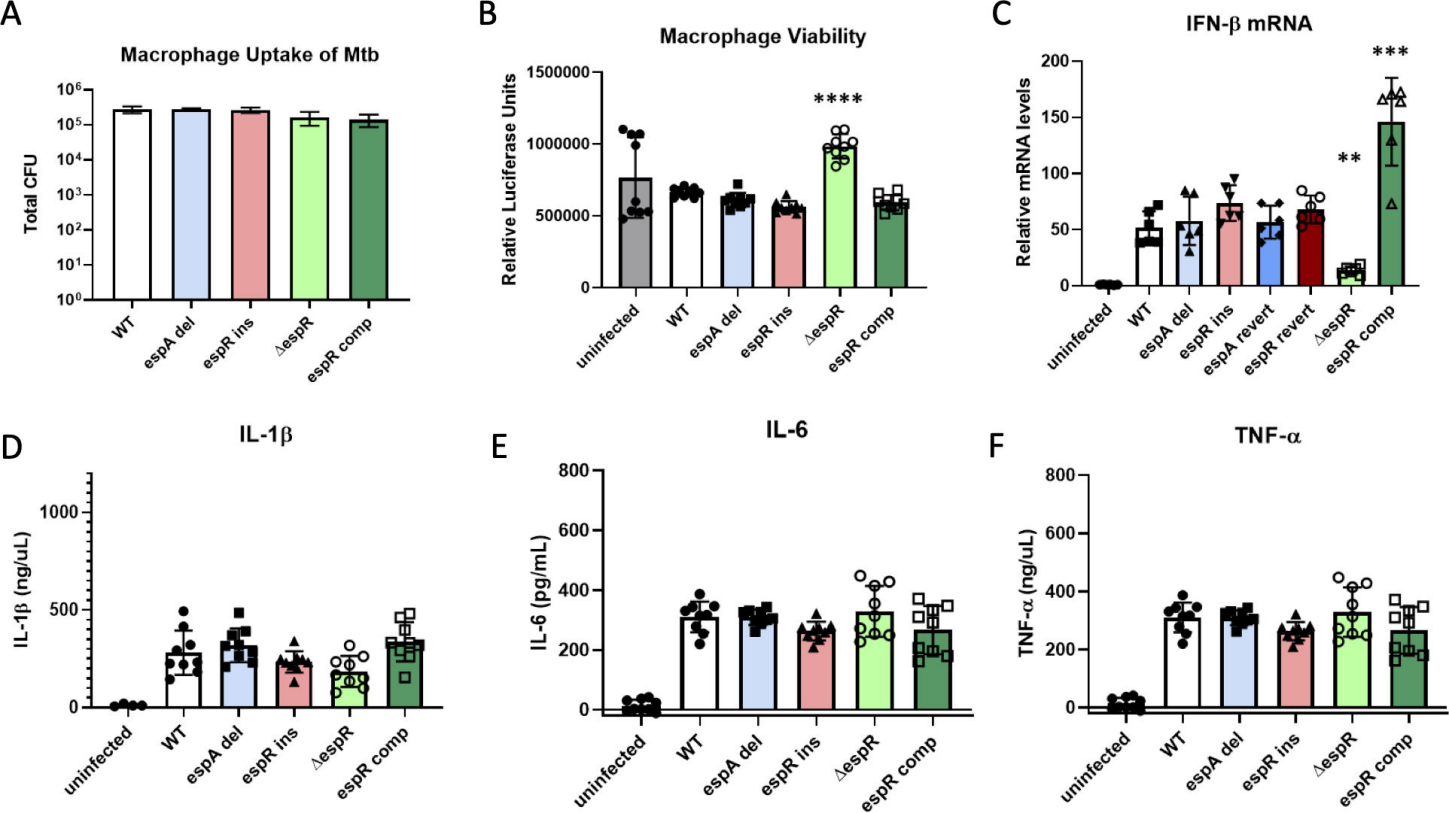
ESX-1 associated homopolymeric indels do not detectably alter macrophage cytokine profiles or cell death. For all macrophage experiments, BMDMs were infected with Mtb at a theoretical MOI of 9. (**A**) Four hours post-infection cells were lysed and plated for CFU. Three biological replicates are shown with mean and standard deviation indicated. (B) Twenty-four hours post-infection, macrophage viability was assessed using cell titer glo. Mean and standard deviation of nine replicates pooled across two independent experiments are shown. (C) Four hours post-infection, macrophage RNA was harvested. qPCR was performed on IFN-β, using *actB* as an endogenous control. Mean and standard deviation of 6 biological replicates pooled from two independent experiments shown. (D, E, and F) Cell supernatant was harvested 24 h post-infection and ELISA was performed to quantify IL-1β, IL-6, and TNF-α respectively. Mean and standard deviation of 9 replicates pooled across two independent experiments are shown. All statistics were performed using ANOVA followed by Dunnett’s multiple comparison testing. *P*-value: *, <0.05; **, <0.01; ***, <0.001; ****, <0.0001.

Aside from antibiotic exposure, the other major selective pressures that may be contributing to the evolution of ESX-1-related variants are derived from the pathogen’s mammalian host. Two major host cellular functions that ESX-1 impacts are macrophage cell death and cytokine production. We sought to test whether either *espA*- or *espR*-associated variants impacted these functions in murine bone marrow-derived macrophages (BMDM). *EspR* knockout and complemented strains were included as positive controls. We first measured bacterial colony-forming units (CFU) 4 h after infection at an MOI of 9, and found no significant differences in bacterial uptake between strains (Fig. 6A). We were also unable to detect any differences in macrophage viability at 24 h post-infection between either of the ESX-1 indel strains; however, we observed a significant increase in macrophage viability in cells infected with ∆*espR*, which was reversed by complementation (Fig. 6B).

Macrophage type-I interferon-regulated genes are induced by Mtb in an ESX-dependent manner (53). To test whether *espA* or *espR* indel mutants resulted in changes in interferon-β production, we harvested RNA from BMDMs following 4 h of infection, when interferon-β transcripts are at their peak (53). The observed induction of interferon-β upon infection was largely abrogated by deleting *espR*, demonstrating that our assay was sensitive to *espR*-dependent effects. However, we were not able to detect any significant differences in transcript levels between the wild type and the homopolymer indel mutants (Fig. 6C). To further characterize the impact of these mutations on host-pathogen dynamics, we harvested cytokines from BMDMs 24 h post-infection. Neither TNF-α, IL-1β, nor IL-6 secretion was significantly impacted by any of the mutations we introduced (Fig. 6D through F). Thus, while the cell death and IFNβ assays were sensitive to complete *espR* deletion, we were unable to detect an effect of the less penetrant *espA* or *espR* homopolymer indels on these responses.

### ESX-1 homopolymer mutations increase fitness in a mouse infection model

To determine whether ESX-1 related homopolymer indels altered interactions with the host in the more complex infection environment of the intact animal, we quantified the effect of these mutations on bacterial fitness in the mouse lung. ESX-1 fulfills two potentially contrasting functions, by promoting intracellular growth while also secreting major T cell antigens. To separate these potential functions, the relative fitness of each mutant in both wild-type and T-cell deficient mice was determined.

C57BL/6 or TCRα deficient mice were infected via aerosol with a pool of DNA-barcoded Mtb strains (Table S3). This competitive infection model was used to reflect the natural setting in which relatively rare HT variants are selected. To control for possible fitness effects of different antibiotic markers or second site mutations, we independently isolated multiple wild-type and mutant clones that contained the same markers, and generated a small pool of strains containing two independently derived and barcoded clones of WT, *espA*-del, and *espR*-ins strains. Additionally, this pool included a single barcoded strain lacking EccB1, an ATPase and core component of ESX-1 secretion machinery (54), to model complete ESX-1 deficiency (44).

**Fig 7:**
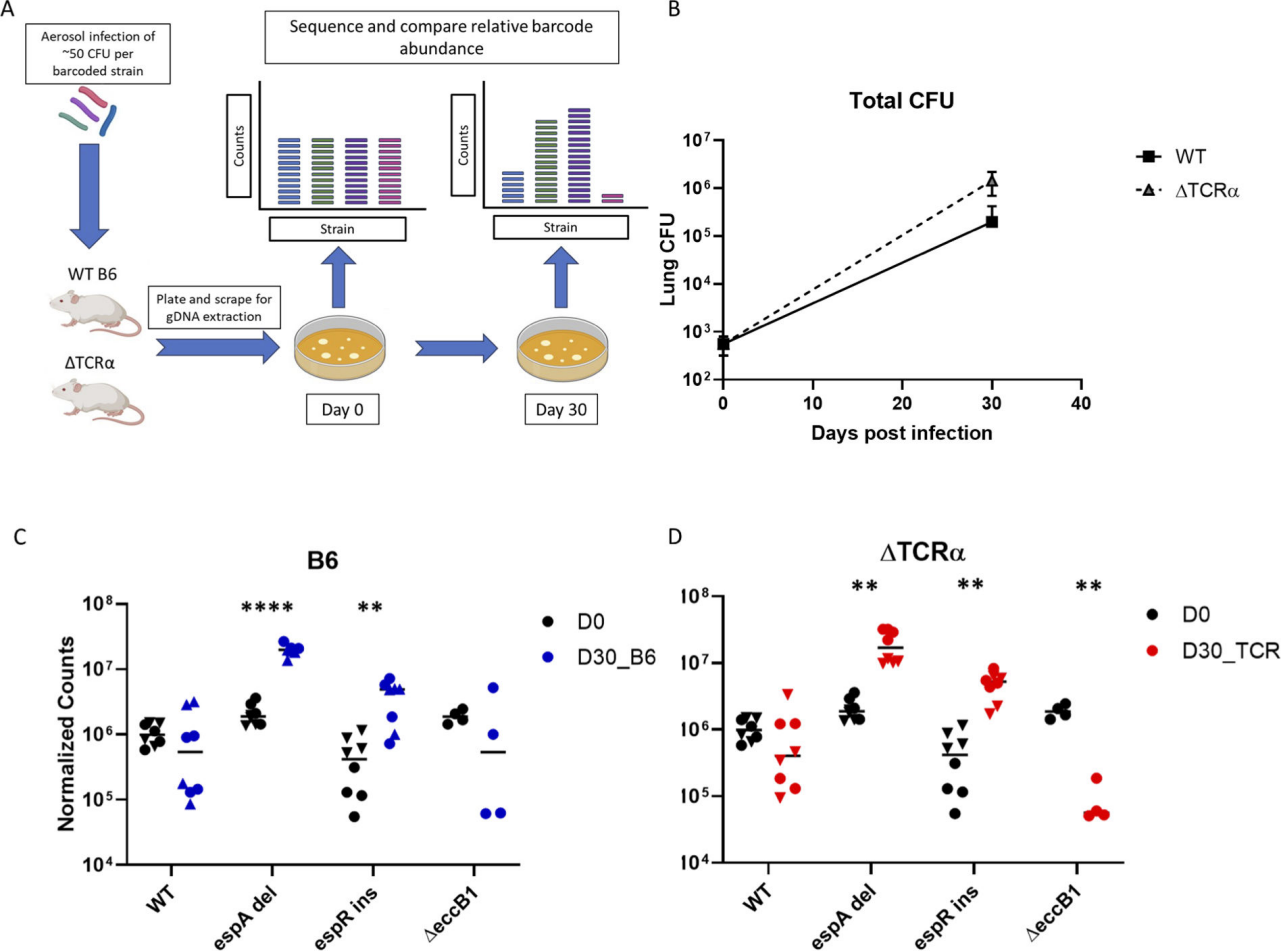
ESX-1 mutants show fitness advantage during mouse infection in a T-cell independent manner. (A) Schematic showing experimental design of pooled infection of barcoded Mtb strains (total CFU ~ 400). (B) Lung CFU at day 0 and day 30 from WT or ∆TCR-α mice aerosol infected with minipool. Mean and standard deviation from each timepoint plotted. (C and D) Normalized sequencing counts of barcodes from mouse lung homogenate at day 0 and day 30 in (C) WT B6 and (D) ∆TCR-α mice. Two independently derived barcoded WT, *espA*, and *espR* strains and one ∆*eccB1* were pooled for analysis. Different point shapes represent individual barcodes for each strain from four different mice with median plotted. Two-way ANOVA followed by Dunnett’s test for multiple comparisons was performed on paired barcode counts at D0 and D30 for each genotype. *P*-value: *, <0.05; **, <0.01; ***, <0.001; ****, <0.0001.

Mice were infected with a pool containing ~50 CFU of each of the infecting strains (Fig. 7A). On day 0 and day 30, mice were sacrificed and lung homogenate was plated to recover surviving bacteria. After 30 days of infection, total bacterial burden in the lung reached 1.99 × 10^5^ in wild-type mice and 1.45 × 10^6^ in TCRα deficient mice (Fig. 7B). The strain-specific barcodes were amplified from bacterial genomic DNA, deep sequenced and quantified. The relative proportions of each strain were then determined by normalizing to their abundance on day 0 such that relative WT counts remained constant, and then comparing relative abundances at D0 to those at D30 for each genotype. In all cases, the independent mutants corresponding to each genetic variant behaved similarly, and all data points were included in the statistical testing. The mean relative abundance of the ESX-1 deficient ∆*eccB1* mutant was lower than wild type in both mouse strains, reaching statistical significance in the TCRα-deficient animals. In contrast, both of the clinically prevalent mutations produced a selective advantage when compared to wild-type Mtb, and this phenotype was unaffected by the presence of T-cells (Fig. 7C and D). The fitness advantage in C57BL/6 mice conferred by *espA-*del and *espR-*ins on a per generation basis was 44% and 18%, respectively. These data are consistent with the positive selection on the *espR*-ins and *espA-*del variants that were inferred from phylogenomic analysis of clinical isolates.

## DISCUSSION

The adaptability of Mtb to different environments and epidemiological situations contrasts with the stable genome sequence of the organism. This study takes a step towards resolving this paradox, demonstrating that homopolymer-dependent phase variation may provide a mechanism for Mtb to strategically direct genetic variation to certain sites, particularly those that result in differential fitness in the context of alternating pressures. ESX-1 associated genes are particularly attractive targets for such variation given the central role this system plays in promoting intracellular growth, maintaining the integrity of the cell wall, and contributing to the production of immunodominant antigens (36, 39, 40). The opposing pressures resulting from these activities must be balanced by Mtb in different contexts, and the consequences of tuning this system depend upon the specific pressures that the bacterial population encounters. As such, we posit that tuning ESX-1 components up or down throughout infection may have differential fitness effects within different niches or at different timepoints. This may explain why natural variants resulting in both up- and downregulation of ESX-1 components remain positively selected, despite the opposing directionality of their effects on ESX-1 function. We also note that EspR has effects beyond regulating the *espACD* operon that could play a role in tuning fitness (45).

In this study, we demonstrate that both host and drug pressures may act to select ESX-1 related homopolymer indel variants. These individual effects, as well as the interaction between them, may be important determinants of clinical outcome. The effect of the *espR*-ins mutation on INH sensitivity is consistent with previous reports showing that disrupting *espR* function by either CRISPRi or transposon mutagenesis results in a decreased isoniazid MIC *in vitro* as well as an increased rate of bacterial killing by the drug in a mouse model (27, 55). While the effect of *espR*-ins on INH sensitivity was relatively modest (0.039 µg/mL to 0.079 µg/mL), even smaller INH MIC changes have been associated with increased risk of relapsing infection (56). Paired with the fact that most relapses are caused by a strain that does not initially display high level drug resistance (57), this suggests the potential importance of common variants, such as *espR* homopolymer indels, that decrease drug efficacy but are not apparent in standard drug sensitivity testing (47). Seven-base-pair HTs are highly mutagenic in mycobacteria, producing indel frequencies as high as 3.8 × 10^−5^ indels/CFU in a bacterial population (29). Thus, it is likely that these variants are present during infection and could emerge during therapy. This scenario is particularly likely if the *espR* variant is also under selection by host immune pressures prior to therapy. We can only speculate on the specific mechanisms that underlie the increased fitness of ESX-1 related homopolymer indel mutants. Our transcriptional profiling data show that both *ahpCD* and *inhA* are increased in the *espR*-ins strain, providing a possible mechanism for altered INH sensitivity. As the increased fitness of these strains in mice appears unrelated to T cell recognition, we suggest that alterations in a cell-autonomous interaction with lung phagocytes may underlie this effect.

One notable characteristic of the *espR*-ins mutation is its location within an extended 5′UTR. Our studies indicate that this 5′UTR represses the translation of EspR, and that this regulation is relieved by the *espR*-ins phase variant. Extended 5′UTRs often act as *cis*-regulatory elements in bacteria by altering translation of the mRNA (58). Some 5′UTRs can act as riboswitches that sense the presence of a ligand and alter protein production in response to environmental cues, while others interact with regulatory RNAs (58–60). Notably, the B11 small RNA in *Mycobacterium abscessus* was recently described to regulate ESX-4 (61), a functionally analogous secretion system to ESX-1 that also contributes to bacterial virulence (62). Future investigations may shed light upon whether the *espR* 5′UTR responds to a similar small RNA or other cues to regulate EspR function.

Here we describe two variable homopolymers in ESX-associated loci, but there may be more such sites of phase variation that impact ESX function. We previously also found a highly variable homopolymer within the coding body of another ESX-1 component, *espK*, and other studies have also found signatures of reversible selection within ESX-1 genes, notably *espI*, providing further evidence that this system may be subject to alternating pressures (33, 63). Although this study investigated how host and drug pressures may have selected for certain ESX-1 associated variants, it is important to note that other metrics, especially those related to transmissibility, were not captured in our experiments. Additionally, our study does not explain potential epistatic effects between variable homopolymers, or any lineage-specific effects related to these variants. Of particular interest is the association between *espR* homopolymer indels and the RD236a deletion, which results in the removal of two EspR binding sites upstream of the *espACD* operon. Previous work has demonstrated the importance of cooperative binding sites upstream of *espACD* to facilitate EspR binding (48, 64), particularly among the two more distal EspR binding sites (48), and that an inability to bind multiple EspR sites simultaneously abrogates the ability of EspR to form DNA bridges upstream of *espA* (65). We speculate that removing these two distal binding sites may decouple *espA* activity from the numerous other functions of the EspR regulon, thereby changing the adaptive landscape at the *espR* locus. This scenario is reminiscent of the epistatic interaction between single nucleotide variants in the *espACD* regulator, *phoPR*, and the RD8 deletion that is also upstream of *espACD* (42). Further study may be warranted to investigate the impact of layering ESX variants upon each other, or whether certain kinds of cryptic variation only reveal themselves in specific genetic backgrounds.

We have shown that single base pair insertions and deletions at HTs in regulatory regions of ESX-1 associated genes can influence transcriptional patterns and impact how Mtb interacts with both host and drug pressures. This research supports the idea that Mtb must strike a balance between opposing evolutionary forces to thrive in the face of the many drug and immune pressures it is subjected to. Since Mtb does not acquire new DNA sequences via horizontal gene transfer, homopolymer-dependent contingency loci provide a mechanism for Mtb to adapt to alternating pressures and remain fit for the various contexts the bacterium encounters across the globe. Overall, this study suggests the potential wealth of genetic variation that may be encoded throughout the Mtb genome in HTs, enabling Mtb to adapt to the various alternating pressures it faces throughout its life cycle.

## MATERIALS AND METHODS

### tSNE generation

Mtb isolates (*n* = 26,903) from four lineages were plotted as previously described (33). RD236a region was determined based on sequencing. Isolates with an average depth of less than 0.1 mapping to the RD236a region (NC_000962.3:4056945–4058396) were deemed to have the region deleted. RD236a deletions were validated using Delly as described by reference 66.

### Phylogenetic tree construction

A core SNP-based phylogenetic tree of 933 publicly available lineage one strains was created using RAxML-ng (v1.2.1) under the GTRgamma substitution model e- with 200 bootstrap replicates with *Mycobacterium canetti* used as an outgroup.

### Mtb media and growth conditions

*M. tuberculosis* H37Rv was grown in 7H9 broth with 0.05% Tween 80, 0.2% glycerol, and OADC; transformants were selected on 7H10 plates with 0.5% glycerol and OADC.

### Strain generation

*espR*-ins mutant was generated as previously described (33). Electroporation was performed using 2 µg espR target oligo CGTCGTGTGGACACATCACCGAATCGGTTGGACCCTCATCGGGGGGGGTCTTCGTTGACCCCTCACAACGTCAGCACCCAATCCGCTC and 200 ng hygR repair oligo CGGTCCAGCAGCCGGGGCGAGAGGTAGCCCCACCCGCGGTGGTCCTCGACGGTCGCCGCG.

Candidate clones were amplified using the primers CAAAGGAGGCTCTCGGCGAATC and GTCGATCCGGTCCAACACCTTC. The PCR product was sequenced with CAAAGGAGGCTCTCGGCGAATC.

To generate revertants, cells were electroporated using 2 ug of target oligo encoding wild-type sequence, along with 200 ng of *rpsL* targeting oligo GCGGGCAACCTTCCGAAGCGCCGAGTTCGGCTTCCTCGGAGTGGTGGTGTACACGCGGGTGCATACACC and plated on 7H10 containing 20 µg/mL streptomycin.

∆*espR* was generating using ORBIT, as described previously (67). The gene deletion was targeted using the oligo ACGACGTTCGCTGCCCGCCTGAACCGCCTGTTCGACACGGTTTATCCGCCCGGACGCGGGCCACATACCTGGTTTGTACCGTACACCACTGAGACCGCGGTGGTTGACCAGACAAACCCGATGCGCGACGACGGCGTGCGCCGGATCGCGCAGCGGGCCCACGGGTTGCCCTCCGCGGCGCAGCAGAA and confirmed with the PCR primers GTTTGCTCTTGGAGCCCGG and CTAAGCGTCGATCCCTTCGG.

Complementation of the ∆*espR* mutant and overexpression of EspR in WT Mtb was performed by cloning the *espR* open reading frame (including 285 base pairs upstream of the start site) into pKP1225 (a streptomycin resistant derivative of pMV306 68] that integrates at the phage L5 site [69]) and selecting Mtb transformants on 20 µg/mL streptomycin. Plasmids were amplified and sequenced for confirmation with the primers GACCATGATTACGCCAAGCTC and CAATTCGTTCAAGCCGACGC.

### RNA extraction and sequencing

RNA extraction and sequencing were performed as previously described (33) and processed on Illumina HiSeq 4,000 with a 2 × 150 base pair sequencing configuration. The reads were mapped to the H37Rv genome (GenBank accession NC_000962.2) using BWA (v0.7.12). DeSeq (70) was used to analyze the counts and identify differentially expressed genes as genes with an adjusted *P*-value < 0.05.

### Mass spectrometry

Detailed mass spectrometry methods are provided in Supplemental Methods.

### qPCR

qPCR performed using a ViiA 7 QPCR RT-PCR machine and New England Biolabs Luna Universal One-Step RT-qPCR Kit. Each biological replicate was performed in technical triplicate, the average of which was used to represent each biological replicate. *espA* levels were quantified using the primers GCTGATCAACGCGACTCAAC and TGAACTCCCACACTTCTCCG. Samples were normalized to the amount of *sigA. sigA* levels were measured using primers CCGGTGATTTCGTCTGGGAT and ATCTGTTTGAGGTAGGCGCG.

For measuring mRNA levels of luciferase reporter, *sigA* was used to normalize. Primers used to measure reporter mRNA levels were as follows: CTGCACTACGGTACTCTGGTC and CTTGTTGCCGTTCCACAACG.

For qPCR of macrophage RNA, IFN-β was quantified as reported by Ji et al. (71).

### RNA folding prediction

*espR* UTR sequence with or without homopolymer expansion was loaded into Vienna RNAfold webtool with standard settings (72).

### Translational reporter assay

The 142 bp 5′ *espR* UTR (24 bp for no-UTR construct), driven by a p16 promoter (33), was fused to the codon-optimized nano-luciferase gene by Gibson assembly in an MCtH vector (73) digested using MfeI and NdeI. Strains were grown to an OD of 1.0 prior to assaying luciferase activity using Promega Nano-Glo Luciferase Assay.

### Antibiotic MIC determination

Bacteria grown to mid-log phase were added to a 96-well plate at an OD of 0.05. All conditions were prepared in triplicate. Isoniazid and rifampicin concentrations began at 0.5 and 1 µg/mL, respectively, and serially diluted twofold. Ethambutol, linezolid, bedaquiline, and moxifloxacin concentrations began at 100, 10, 1, and 10 µg/mL respectively, and serially diluted 2.5-fold. Minimum inhibitory concentrations were calculated by nonlinear regression generating IC50 values while constraining all other parameters.

### Macrophage infections

Bone marrow derived macrophages were isolated from C57BL/6 mice as previously described (74) and infected with Mtb at a theoretical MOI of 9. For CFU plating, cells were lysed with 1% Saponin/PBS and serially diluted onto 7H10. For RNA harvesting, Zymo Research Direct-zol RNA Miniprep Plus Kit was used. After 24 h, macrophage viability was profiled using Promega CellTiter-Glo 2.0 Cell Viability Assay.

### Cytokine measurements

Cell supernatants were harvested at 24 h and quantified using R&D Systems DuoSet ELISA kits for IL-6, IL-1 beta, and TNF-alpha.

### Mouse infections

C57BL/6 J mice were purchased from The Jackson Laboratory. TCRα KO mice, also from Jackson Laboratory, were a gift from the Behar Lab. Mice (8–10 weeks old) were infected with pooled strains at equal ratios by aerosol at a total CFU of ~400 per mouse. On days 1 and 30, mice were euthanized and lungs harvested and homogenized before plating on 7H10 with 20 µg/mL streptomycin. Plates were scraped and gDNA was harvested as described previously (75).

### DNA sequencing and barcode analysis

Sequencing libraries were prepared by amplifying barcode region using primers that bind common sequence flanking the barcode, similar to described previously (31). Sequencing was performed on NextSeq2000 using P1 Reagent 100 cycle kit.

To estimate strain abundance, we generated a FASTA file containing each strain and barcode, building reference index files using Subread-buildindex (76). FASTQ files were aligned to indexed reference files using subread-align. BAM files were sorted and indexed, and the number of unique reads aligned to the barcodes was determined using Samtools idxstats (77). Barcode counts were first normalized across all samples by multiplying each sample by a normalization factor derived from dividing the total number of aligned barcode reads from the mouse with the largest read count by the number of reads from each individual mouse (reads_max_/reads_sample_). A second round of normalization was performed on D30 reads by multiplying all barcode counts by the ratio of WT counts at D30 to WT counts at D0, such that all changes in barcode abundances would reflect a competition index versus the WT strains. These normalized abundances were then compared between D0 and D30 for each genotype.

## SUPPLEMENTAL MATERIAL

Supplemental Methods mbio.03762-24-s0001.docx Description of targeted mass spectrometry methods.

Table S1 mbio.03762-24-s0002.xlsx RNAseq comparing espR-ins to WT H37Rv.

Table S2 mbio.03762-24-s0003.xlsx RNAseq comparing ∆*espR* to WT H37Rv.

Table S3 mbio.03762-24-s0004.xlsx Unique barcode identifiers and sequencing primers used for mouse competition sequencing experiments.

ASM does not own the copyrights to Supplemental Material that may be linked to, or accessed through, an article. The authors have granted ASM a non-exclusive, world-wide license to publish the Supplemental Material files. Please contact the corresponding author directly for reuse.
