# Supplementary material for: Frequently arising ESX-1-associated phase variants influence Mycobacterium tuberculosis fitness in the presence of host and antibiotic pressures
Source: mBio. 2025 Jan 28;16(3):e03762-24. doi: 10.1128/mbio.03762-24 (PMC11898584; doi:10.1128/mbio.03762-24)
Supplement: Supplemental Methods — Description of targeted mass spectrometry methods. [file mbio.03762-24-s0001.docx]

**Detailed Mass Spectrometry Methods**

**Sample preparation**

Mtb cultures were grown up to an OD of ~0.4 before being washed 2x with phosphate-buffered saline with 0.05% Tween-80 to remove protein contaminants from the supernatant. The cells were resuspended in albumin-free 7H9 containing 0.05% Tween 80, 0.2% glycerol, and 0.2% dextrose and allowed to grow for 36 more hours. Supernatants were harvested and concentrated using Amicon® Ultra Centrifugal Filter, 3 kDa MWCO. Lysates were resuspended in sterile 1X PBS containing Roche cOmplete EDTA-free protease inhibitor cocktail and transferred to the MPbio 0.1 mm silica bead tubes for bead beading 4x with an MPBio FastPrep Bead-Beater. SDS was added to a final concentration of 2% prior to heating and vortexing the solution and harvesting lysate.

The cell samples were flash frozen on dry ice and subsequently dried using a Speed-Vac. Next, 23 µl of 1x lysis buffer was added to the protein pellet, initiating the Suspension trap (S-Trap) protocol. The proteins underwent reduction by 10mM TCEP and alkylation by 20mM Iodoacetamide. Following an overnight digestion at 37°C, 40 µL of 50 mM TEAB elution buffer was added and spun at 4000 rpm for 1 minute. This was followed by a 1-minute spin with 40 µL of 0.2% Formic acid (FA) and, lastly, 40 µL of 50% acetonitrile (ACN). The elution was then pooled together and dried using a Speed-Vac.

The digested samples were reconstituted with 20 µL of 0.1% Formic acid in 5% acetonitrile, followed by centrifugation at 16000 rcf for 15 minutes. Subsequently, 18 µL of the sample was transferred to non-binding HPLC vials.

**LC-MS/MS Analysis**

The samples (4 µL injection) were analyzed on a TimsTOF Pro2 (Bruker) mass spectrometer, which was coupled to a nanoElute LC system from Bruker. For all analyses, a Bruker Fifteen Pepsep column (150 mm x 75 μm id, 1.9 μm, 120 Å pore size) was utilized. A 60-minute gradient was employed for DDA-PASEF and a 30-minute gradient used for DIA-PASEF analysis, with a flow rate set at 500 nL/min. The captive nano-electrospray voltage was maintained at 1600V, using one column configuration (no trap) and a solvent composition of Solvent A: 0.1% FA in water and Solvent B: 0.1% FA in ACN.

*DDA-PASEF method*: The method consists of 10 MS/MS PASEF scans per topN acquisition cycle, with ramp and accumulation times of 100 ms, covering an m/z range from 100 to 1700 and an ion mobility range (1/K0) from 0.70 to 1.30 V s/cm^2. Collision energy settings followed a linear function of ion mobility, ranging from 20 eV at 0.6 V s/cm^2 to 59 eV at 1.6 V s/cm^2, using default parameters. Calibration of the instrument was performed using three ions from the ESI-L Tuning Mix (Agilent) (m/z 622, 922, 1222).

**Data analysis workflows**

*Fragpipe/MSFragger/ScaffoldQ+S DDA label free quantitative analysis*

The custom workflow ‘LFQ-MBR’ from FragPipe version 21.1 was used, including database search with MSFragger (version 4.0), deep-learning prediction rescoring with MSBooster, Percolator and ProteinProphet (Philosopher version 5.1.0) for PSM validation and protein inference. The raw .d files from Bruker were searched against a *Mycobacterium tuberculosis* (UP000001584) isoform fasta database appended with common contaminant proteins, containing in total 3995 sequence entries. Decoy reversed sequences were appended to the search database. The default MSFragger search parameters were used, except precursor and fragment mass tolerances were set to 20 ppm; enzyme: trypsin (not cutting before P); peptide length 7-30. Variable modifications were set to oxidation of Met and acetylation of protein N-t, and fixed modifications to carbamidomethylation of Cys. MSBooster, Percolator and ProteinProphet default options were used, and results were filtered by 1% FDR at protein level. The searched results were then imported to Scaffold Q/S 5.2.2 for data visualization and statistical analysis. For EspR, the following peptides were quantified: AAYFTDDEYYEK, AAYFTDDEYYEKLDK, and GPHTSAEVIAALK. These total precursor intensities were then compared relative to SigA peptides: AKNHLLEANLR, NHLLEANLR, ELLQDLGREPTPEELAK, VALLNAEEEVELAK, and VALLNAEEEVELAKR. EspA and CFP10 peptides in the supernatant were quantified using the following peptides: EspA: APVEADAGGGQK, APVEADAGGGQKVLVR, NHVNFFQELADLDR, QLISLIHDQANAVQTTR, YSEGAAAGTEDAER, and YSEGAAAGTEDAERAPVEADAGGGQK; CFP10: FQEAANK, FQEAANKQKQELDEISTNIR, GAAGTAAQAAVVR, GAAGTAAQAAVVRFQEAANK, ISGDLKTQIDQVESTAGSLQGQWR, QELDEISTNIR, QKQELDEISTNIR, TDAATLAQEAGNFER, and TQIDQVESTAGSLQGQWR. Both proteins quantified in supernatant were compared to Ag85b levels using the following peptides: AGCQTYK, GDLQSSLGAG, LVANNTR, NDPTQQIPK, NDPTQQIPKLVANNTR, VQFQSGGNNSPAVYLLDGLR, and WETFLTSELPQWLSANR.
